# Mean platelet volume and the association with all-cause mortality and cardiovascular mortality among incident peritoneal dialysis patients

**DOI:** 10.1186/s12872-023-03551-x

**Published:** 2023-11-08

**Authors:** Jianghai Wang, Xiaochen Ma, Xuepeng Si, Mingyang Wu, Wang Han

**Affiliations:** 1 Emergency Department of Dongying People’s Hospital, Dongying, 257091 China; 2 Obstetrics Department of Dongying People’s Hospital, Dongying, 257091 China; 3 Department of Nephrology of Zhucheng People’s Hospital, Weifang, China; 4 Emergency Department of Dongying People’s Hospital, Dongying, 257000 China

**Keywords:** All-cause mortality, Mean platelet volume, Cardiovascular Disease, Peritoneal dialysis, Cardiovascular mortality

## Abstract

**Background:**

While mean platelet volume (MPV) is linked to severity and all-cause mortality in patients with sepsis, its association with all-cause mortality and cardiovascular mortality in patients treated with peritoneal dialysis (PD) remains unknown.

**Objectives:**

The purpose of this study was to estimate the relationship between MPV and all-cause mortality and cardiovascular mortality among patients treated with PD.

**Method:**

We retrospectively collected 1322 patients treated with PD from November 1, 2005 to August 31, 2019. All-cause mortality and cardiovascular mortality was identified as the primary outcome. MPV was classified into three categories by means of X-tile software. The correlation between MPV and all-cause mortality was assessed by Cox model. Survival curves were performed by Kaplan-Meier method.

**Results:**

The median follow-up period was 50 months (30–80 months), and a total of 360 deaths were recorded. With respect to all-cause mortality, patients in MVP ≥ 10.2 fL had considerably higher risk of all-cause mortality among three models (HR 0.68, 95%CI 0.56–0.84; HR 0.70, 95%CI 0.56–0.87; HR 0.73, 95%CI 0.59–0.91; respectively). Moreover, patients treated with PD, whose MVP ≥ 10.2 fL, also suffered from significantly higher risk of cardiovascular mortality in model 1, 2, and 3 (HR 0.63, 95%CI 0.46–0.85; HR 0.66, 95%CI 0.48–0.91; HR 0.69, 95%CI 0.50–0.95; respectively).

**Conclusions:**

This study indicates that MPV is independently correlated with both all-cause mortality and cardiovascular mortality in PD.

## Introduction

Mean platelet volume (MPV) is an inflammatory marker and a biomarker in the routine blood count report, which is also a signaling molecule for both the stimulation and function of platelets. It frequently serves as a gauge for platelet size and provides information on how quickly platelets are produced and activated [1]. In those previous studies, MPV had been associated with an increased incidence of hypertension [2]. And the latest studies found that MPV can be a predictor of sepsis severity and mortality, as well as platelet count recovery in dengue patients [3]. In the process of continuous studies, a series of cancer’s relevant studies, such as prostate cancer, oral cancer and lung cancer, are closely related to MPV. Muhyettin Omar et al. found that the role of MPV is poor prognostic factors in lung cancer [4]. Ghazi Abuzaid suggested that MPV could be meaningful prognostic predictors for low survival rates [5]. The normal value of MPV is 7–11 (fl.), and its change is affected by the status of hematopoietic function of bone marrow, diabetes mellitus, myocarditis and other factors [6]. The value of MPV is important for the clinical diagnosis of patients.

Cardiovascular disease (CVD) is a set of heart and vascular diseases always represented by coronary heart disease (CHD), congestive heart failure (CHF), angina pectoris, as well as stroke [7]. MPV played a crucial role in cardiovascular homeostasis [8]. A cohort study indicated that incident patients treated with peritoneal dialysis (PD) possessing greater platelet (PLT) and plateletcrit (PCT) levels may also have a higher risk of cardiovascular mortality [9]. PD includes continuous ambulatory peritoneal dialysis (CAPD) and continuous circulating peritoneal dialysis (CCPD). Mangalachulli Pottammal Ranjith et al. found that in myocardial infarction (MI) patients, MPV may be linked to worse outcomes [10]. However, the relationship between MPV levels and cardiovascular mortality among incident patients treated with PD is unclear.

Although there have been several successful publications in MPV related fields, the relationship between MPV and all-cause mortality and cardiovascular mortality in peritoneal dialysis patients is unclear due to the lack of specific mechanism studies. Therefore, the intention of this research was to investigate the probable link between MPV and all-cause, cardiovascular mortality among incident peritoneal dialysis patients.

## Methods

### Study population and data collection

From November 1, 2005, to August 31, 2019, all patients who got selected have had PD renal replacement therapy at the PD Center, belonging to dongying people’s hospital. The criteria for inclusion were as follows: (1) with an age of more than 18 (2) accepting the treatment of peritoneal dialysis (3) living for 90 days or more after receiving the treatment (4) residual kidney function and weakly total (Kt/V) can still support normal life activities. Those who had any of the following characteristics were excluded: (1) under 18 years of age (2) shifted from hemodialysis or an unsuccessful kidney transplant (3) ceased receiving peritoneal dialysis during the first three months of therapy and patients with acute infection within three months of peritoneal dialysis treatment or had other chronic inflammation (4) missing Mean platelet volume data (5) known malignancy, persistent renal and an autoimmune condition (6) patients with rheumatic disease, liver cirrhosis or consuming immunosuppressor medicine. The study was authorized by the Human Ethics Committee of the Sixth Affiliated Hospital of dongying people’s hospital (20,221,067).

The patient follow-up was terminated upon the discontinuation of PD, failure to following up, death, or December 31, 2020. Patients’ death is defined as myocardial ischemia or infarction, congestive heart failure and sudden cardiac arrest. The demographic information of patients included sex, age, and prehospital comorbidities, including premorbid CVD and diabetes. The crucial clinical and biological variables to consider were as follows: body mass index (BMI), Charlson Comorbidity Index (CCI), hypertension, hemoglobin, albumin, total cholesterol (TC), triglyceride (TG), low density lipoprotein (LDL), high density lipoprotein (HDL) and Antiplatelet medications [11, 12].Through the utilization of customary direct procedures, the concentrations of HDL, LDL, and TG were measured [13]. 1.5ml of venous blood was drawn into the corresponding test tube. The Sysmex XE-2100 automatic blood cell analyzer is used for routine blood test, and its supporting reagents, blood cell quality control products and calibration products are provided by Sysmex Japan. Blood samples for laboratory analysis are taken after 8–12 h of fasting. Collecting baseline data regarding demographics and blood sample results 1–3 months after PD happened, and using the creatinine equation of Chronic Kidney Disease Epidemiology Collaboration to evaluate residual renal function.

### Statistical analyses

X-tile is a significant instrument used for outcome-based cutoff point optimization and was applied to pick out the ideal cutoff value of MPV [14]. The patients were split into two groups based on the X-tile method (Yale University, New Haven, CT, USA): low MPV (< 10.2 fL) and high MPV (≥ 10.2 fL). Utilizing percentages and frequencies to represent categorical variables, means as well as standard deviations (SDs) to represent normally distributed data, and medians and interquartile ranges to represent non-normally distributed data. Employing MPV Chisquare, one-way ANOVA, or Kruska-Wallis tests to examine how distinct MPV types differ from one another in terms of continuous variables and classification statistics. In order to generate the survival curve and compare it with the logarithmic rank test, the Kaplan-Meier technique was applied to calculate the cumulative incidence. The relationship between MPV and all-cause mortality was investigated using a Cox proportional hazard model. Moreover, three multivariable proportional hazard models were constructed for this investigation. Multivariate Cox proportional hazard regression was conducted through choosing the clinically prominent covariates and variables with P 0.05 in univariate Cox analysis. Model 1 was unadjusted crude HR. Model 2 was adjusted for age, gender, Charlson comorbidity index, diabetes, hypertension, premorbid cardiovascular diseases, body mass index (BMI) and antiplatelet medication. Model 3 was further adjusted for hemoglobin, albumin, HDL as well as LDL. The possible impacts of age, sex, premorbid diabetes, premorbid hypertension, and premorbid CVD concerning the relationship between MPV and all-cause mortality were assessed in the stratified multivariate analysis. In regards to hazard ratios (HRs) and 95% confidence intervals (CIs), the pertinent results are presented. For data analysis, SPSS 22.0 (SPSS, Inc., Chicago, IL) was applied, and for survival analysis and stratified analysis, R, version 3.6.0 (http://www.r-project.org) was utilized. Additionally, the results are considered statistically significant if P < 0.05.

## Results

### Baseline patient characteristics

**Fig. 1:**
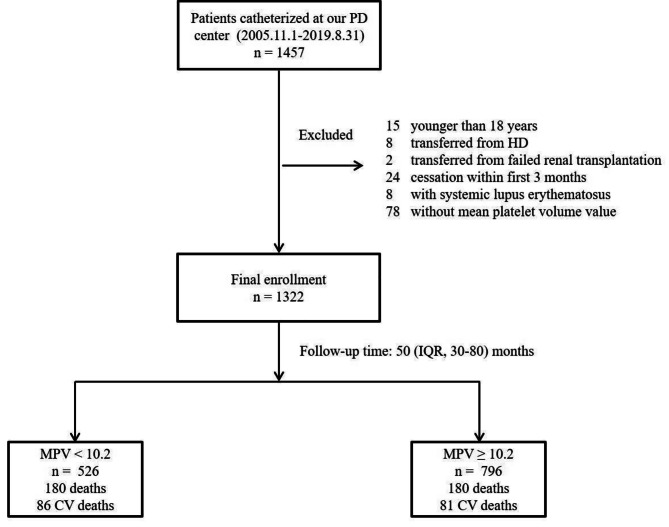
Enrollment flow chart for analysis. PD, Peritoneal Dialysis; HD, Hemodialysis; IQR, Interquartile Range; CV, Cardiovascular; MPV, Mean Platelet Volum

**Fig. 2:**
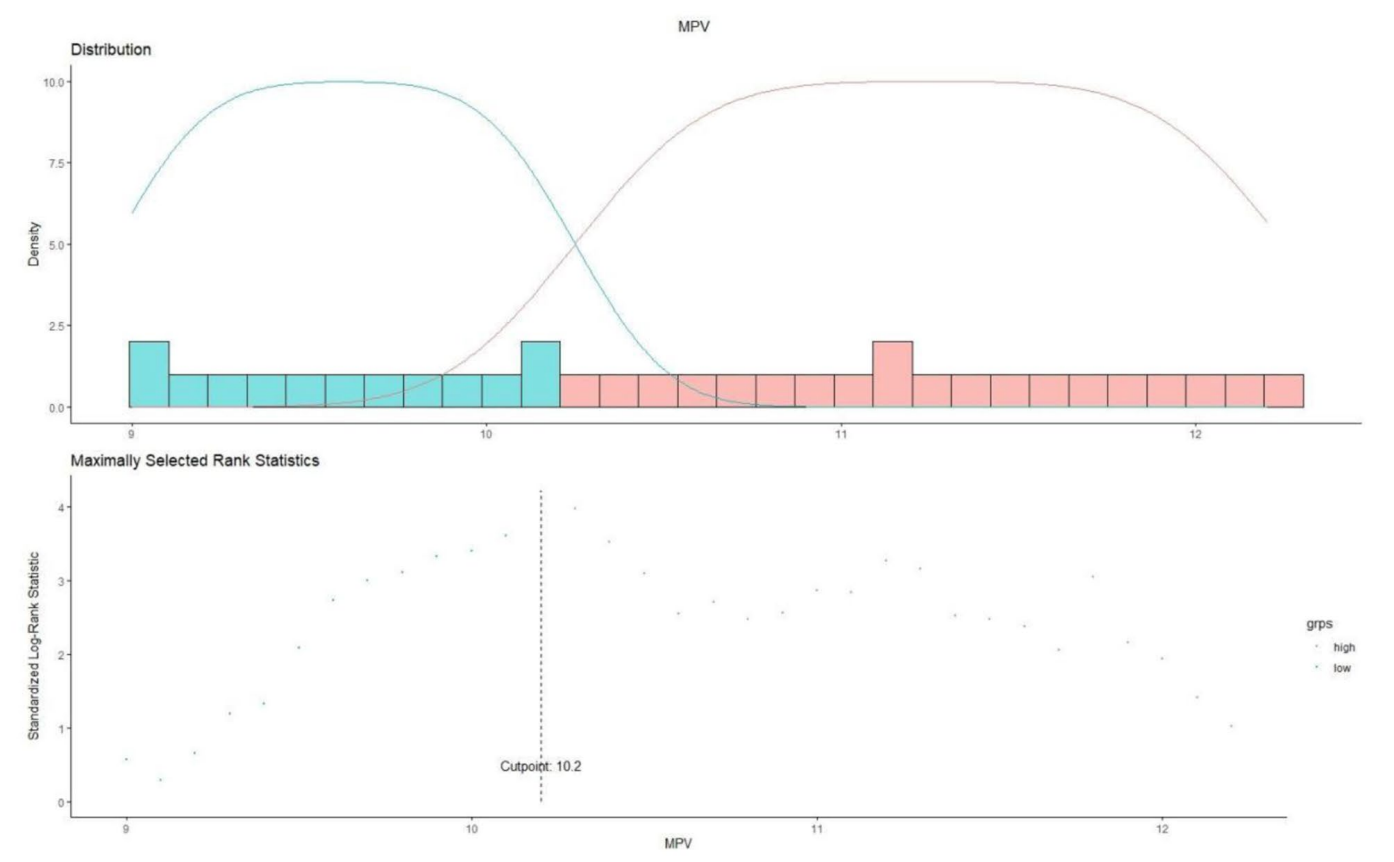
The maximally selected rank statistics for MPV

The records concerning 1457 patients catheterized at our PD center were reviewed. Among these, an aggregate of 135 patients were excluded from the study, including 15 patients who were under the age of 18; 8 patients who were transferred from HD; 2 patients who were moved from failed renal transplantation; and 24 patients ceased within the first 3 months; 8 patients with systemic lupus erythematosus; 78 patients without mean platelet volume value. Therefore, 1322 patients were ultimately registered, and their information was stored for further analysis (Fig. 1). Furthermore, we utilized maximally selected rank statistics to estimate and evaluate the optimal cutpoint for each variable, and finally, the cutpoint of MPV was determined to be 10.2 fL (Fig. 2). A comparison of the baseline characteristics of the enrolled patients is shown in Table 1, of which the mean ± SD age of 49.3 ± 14.5 years and 761 were man (57.6%) were present. There were significant differences in sex, total cholesterol as well as low-density lipoprotein between the two groups (Table 1).

**Table 1:** Characteristics of subjects stratified by mean platelet volume level

Variables	Total (n = 1322)	MPV		*P*-value
		< 10.2 fL (n = 526)	≥ 10.2 fL (n = 796)	
Age (yr)	49.3 ± 14.5	49.0 ± 14.6	49.5 ± 14.4	0.581
Men (%)	761 (57.6)	325 (61.8)	436 (54.8)	0.012
Body mass index (kg/m^2^)	21.8 ± 3.2	21.8 ± 3.1	21.8 ± 3.3	0.985
CCI	2.7 ± 1.1	2.7 ± 1.1	2.7 ± 1.1	0.583
Diabetes (%)	248 (18.8)	97 (18.5)	151 (19.0)	0.839
Hypertension (%)	992 (75.3)	406 (77.6)	586 (73.7)	0.107
CVD (%)	124 (9.4)	44 (8.4)	80 (10.1)	0.312
Hemoglobin (g/L)	80 ± 17	79 ± 18	80 ± 17	0.233
Albumin (g/L)	35.6 ± 5.1	35.3 ± 5.2	35.8 ± 5.1	0.106
Total cholesterol (mmol/L)	4.09 (3.40, 4.85)	4.20 (3.47, 5.01)	4.01 (3.35, 4.74)	0.002
Triglyceride (mmol/L)	1.30 (0.92, 1.81)	1.32 (0.95, 1.83)	1.29 (0.91, 1.79)	0.235
Low-density lipoprotein (mmol/L)	2.33 (1.84, 2.96)	2.36 (1.90, 3.02)	2.30 (1.82, 2.89)	0.042
High-density lipoprotein (mmol/L)	1.09 (0.90, 1.38)	1.10 (0.90, 1.41)	1.09 (0.90, 1.36)	0.356
Antiplatelet medications (%)	66 (5.0)	30 (5.7)	36 (4.5)	0.335

Abbreviations: CCL, Charlson Comorbidity Index; CVD, Cardiovascular Disease. P < 0.05 is considered statistically significant

### The correlation of MPV with all-cause mortality and cardiovascular mortality

In this study, the interquartile range for the follow-up duration was 30–80 months, with 50 months as the median. 360 patients were dead overall at the conclusion of the follow-up, with 167 of those fatalities coming from CVD (46.4%) (Fig. 1). Kaplan–Meier estimates of patients treated with PD with different MPV levels are shown in Fig. 2, which indicated that the participants in the low MPV group (MPV < 10.2 fL) had significantly lower all-cause and cardiovascular mortality (P = 0.00028 and P = 0.0025, respectively).

The associations of MPV with all-cause mortality as well as cardiovascular mortality were examined through the Cox proportional hazard regression (Table 2). The HR (95% CI) of all-cause and cardiovascular mortality for participants in the highest MPV group was 0.68 (0.56–0.84) and 0.63 (0.46–0.85) respectively with unadjusted crude HR in Model 1 in comparison to those in the lowest MPV group; 0.70 (0.56–0.87) and 0.66 (0.48–0.91) respectively with adjustment for age, gender, Charlson comorbidity index, diabetes, hypertension, premorbid cardiovascular diseases, body mass index and antiplatelet medication in Model 2; 0.73 (0.59–0.91) and 0.69 (0.50–0.95) respectively with further adjustment for hemoglobin, albumin, triglyceride, high-density lipoprotein cholesterol and low-density lipoprotein cholesterol in Model 3 (Table 2).

**Table 2:** The associations of MPV with all-cause mortality and cardiovascular mortality using cause-specific hazard models

	Model 1		Model 2		Model 3	
	h (95%CI)	*P* Value	HR (95%CI)	*P* Value	HR (95%CI)	*P* Value
All-cause mortality						
MPV per 1-fL increase	0.93 (0.88–0.98)	0.012	0.93 (0.87–0.99)	0.017	0.93 (0.88–1.00)	0.038
MPV < 10.2 fL	Reference		Reference		Reference	
MPV ≥ 10.2 fL	0.68 (0.56–0.84)	< 0.001	0.70 (0.56–0.87)	0.001	0.73 (0.59–0.91)	0.005
Cardiovascular mortality						
MPV per 1-fL increase	0.88 (0.82–0.95)	0.001	0.88 (0.81–0.96)	0.003	0.88 (0.81–0.97)	0.006
MPV < 10.2 fL	Reference		Reference		Reference	
MPV ≥ 10.2 fL	0.63 (0.46–0.85)	0.003	0.66 (0.48–0.91)	0.012	0.69 (0.50–0.95)	0.024

MPV: mean platelet volume; HR, hazards ratio; CI, confidence interval

Model 1: Unadjusted crude HR

Model 2: Adjusted for age, gender, Charlson comorbidity index, diabetes, hypertension, premorbid cardiovascular diseases, body mass index, and antiplatelet medication

Model 3: Adjusted for age, gender, Charlson comorbidity index, diabetes, hypertension, premorbid cardiovascular diseases, body mass index, antiplatelet medication, hemoglobin, albumin, triglyceride, high-density lipoprotein cholesterol, low-density lipoprotein cholesterol

According to a stratified multivariate analysis, age, gender, diabetes, hypertension and CVD had a profoundly impact on the collaboration between MPV and all-cause mortality. What’s more, higher MPV (MPV 10.2 fL) was correlated with all-cause mortality in males, patients younger than 60 years old as well as patients without diabetes, hypertension, or CVD. Moreover, those who aged < 60, without diabetes and CVD in MVP ≥ 10.2 fL had significantly higher risk of cardiovascular mortality (Fig. 4).

**Fig. 3:**
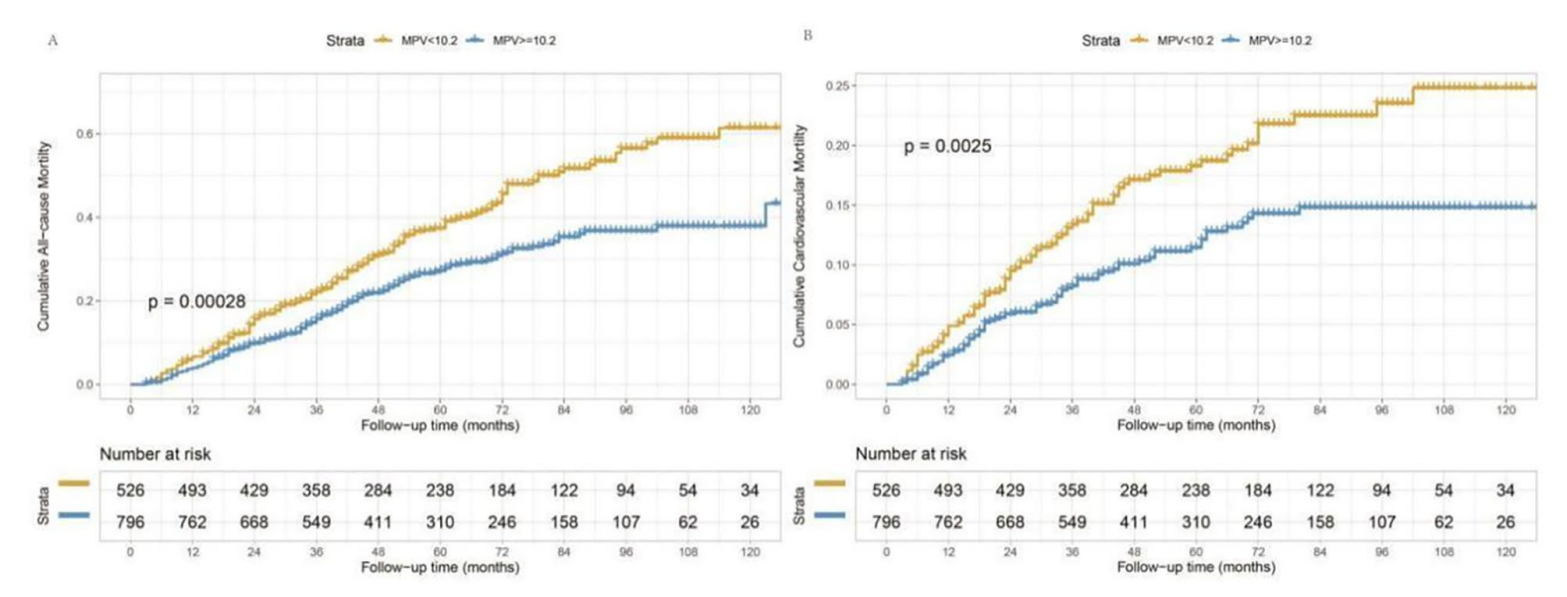
Cumulative incidence of all-cause and cardiovascular mortality for patients treated with peritoneal dialysis stratified by MPV

**Fig. 4:**
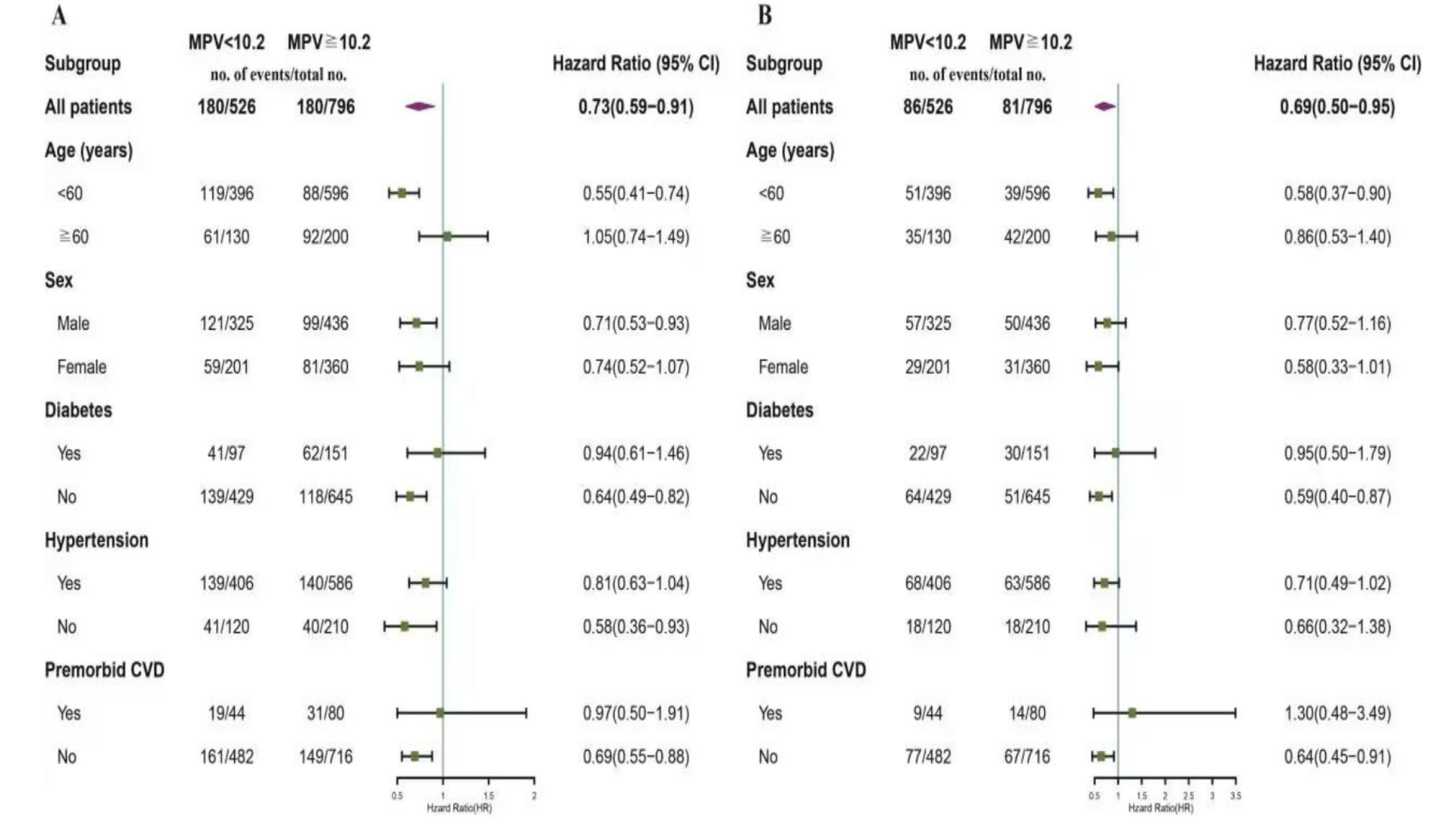
Stratified multivariate analysis of HAR on all-cause mortality and cardiovascular mortality

## Discussion

We discovered a substantial correlation between MPV and cardiovascular and all-cause death in our retrospective cohort analysis. Our findings suggest that high levels of MPV (MPV ≥ 10.2 fL) is an independent hazards and biological marker for all-cause and cardiovascular mortality in patients treated with PD. Based on the most recent search report, this study is the initial study to demonstrate an association between MPV and cardiovascular and all-cause mortality in patients treated with PD.

Some previous typical researches have demonstrated that the levels of several platelet indices, such as plateletcrit, platelet count as well as platelet distribution width, were correlated with mortality in patients receiving dialysis [15]. The link between MPV and mortality in patients treated with PD, however, seemed controversial. Our findings suggested that hypertension has no statistically significant effect on cardiovascular mortality, while in contrast, both Hamdi Pusuroglu et al. and Oh J-Y et al. reported that hypertension had noteworthy correlation with cardiovascular mortality [16, 17]. A previous study from Korea showed that MPV was a prognostic marker in patients with renal damage who needed ongoing kidney replacement therapy [18]. The results of this study were very similar to our results, both indicating that Kaplan-Meier curves showed that patients with MPV ≥ 10.2 fL had a substantially greater 28-day all-cause mortality than patients with MPV < 10.2 fL (P < 0.001). An early systematic review and meta-analysis showed that MPV as a predictor of cardiovascular [19]. In the meantime, recent studies showed that increased MPV are associated with increased risk of mortality in cardiogenic shock patients treated with short-term mechanical circulatory support [20]. Indeed, related studies have confirmed that MPV predicts all-cause mortality and clinical prognostic value in patients with infective endocarditis and patients with sepsis [21, 22]. A recent case-control study also indicated that high MPV values help predict late onset sepsis in preterm infants [23]. In addition, studies by Fayna Rodríguez-González et al. have shown high MPV level is predictors of atrial fibrillation, heart failure and thrombocytopenia [24]. It has to be mentioned that platelet activation is linked with inflammation, thrombosis as well as heart failure. The observation gives rise to the determine that MPV has significant value for the prognostic outcome of many clinical diseases.

As a novel marker to evaluate the danger of all-cause mortality and cardiovascular mortality, MPV were demonstrated playing a role of independent mortality risk factor among hemodialysis, acute ischemic stroke and severe head trauma patients [25–28]. Kim S et al. showed that in comparison to normal MPV, low MPV among hemodialysis patients was linked with a lower mortality risk throughout all multivariable models [28]. In addition, a study by Wu F et al. have shown mean platelet volume-to-lymphocyte ratio (MPVLR) was positively associated with stroke severity and short-term mortality [29]. In febrile neutropenia patients, MPV is the only one complete blood count parameters that substantially connected with 28-day mortality and severe medical complications [30]. However, the special role of MPV in predicting mortality is controversial in other diseases. In patients with ACS treated with invasive therapy, a 3 g/dl reduction in in-hospital hemoglobin levels was common, even in the absence of significant bleeding events, and was independently associated with an increased risk of 1-year all-cause death [31]. Among laryngeal cancer patients, Guo E et al. showed that lower MPV was associated with worse survival [32]. Furthermore, Delago D et al. also found the decreased MPV was associated with poor prognostic in patients with oropharyngeal cancer treated undergoing radiotherapy [33]. Therefore, MPV may be a novel alternative marker of increased risk of mortality, but further relevant research is demanded. In our study, we found that high level MPV (MPV ≥ 10.2 fL) is independently related to all-cause mortality and cardiovascular mortality in patients treated with PD. Nevertheless, the role of MPV among other population, such as patients with peripheral arterial disease is unclear. The current finds further intensify the role of MPV on predicting mortality for other kind of population.

On the one hand, the size and volume of platelets reflect the proliferation and metabolism of megakaryocytes and the formation of platelets in bone marrow. On the other hand, it is closely related to the ultrastructure, enzyme activity and functional status of platelets. with the increase of MPV, platelet aggregation and release function were significantly enhanced, and the all-cause mortality of patients treated with PD was also increasing [34, 35]. The relationship between MVP and cardiovascular and all-cause mortality in patients treated with PD can be accounted for in varieties of ways. Firstly, platelet was demonstrated involving in modulating inflammatory [36].Inflammatory mediators and proinflammatory cytokines released by platelet boost leukocyte recruitment, which induced the release of inflammatory cytokines to continue. In addition, prothrombotic material reserved in platelet promotes platelet activation, platelet adhesion, and vascular neointimal proliferation [36]. Thirdly, glycoprotein Ib and IIb/IIIa adhesion receptors are more densely distributed in platelets and platelets are more reticulated. Therefore, higher level of platelets leads to worse antiplatelet treatment response [37]. The central progress of occlusive arterial disease is the platelet activation and thrombosis and antiplatelet agents ability is charactered by diminishing cardiovascular morbidity and mortality [38]. A meta-analysis conducted by Sansanayudh N et al., revealed that coronary artery disease (CVD) patients with high level of MPV suffered a 16% increased risk of mortality or having a myocardial infarction (MI) than those with low MPV levels [39]. Thirdly, high level MPV may indicate increasing in platelet turnover. Megakaryocytes in the bone marrow release a rising number of youthful, larger, and reactive platelets under conditions of rapid platelet turnover, which lead to a rise in the MPV value. Meanwhile, it has been shown that soluble P-selectin, platelet stimulation indicator, and platelet aggregation are linked to excessive platelet turnover [40]. Doctors and health managers may gain some meaningful implications by our result. Our finding suggests that patients treated with PD with high MPV level (MPV ≥ 10.2 fL) have an increased risk of all-cause mortality and cardiovascular mortality.

There are certain limitations to our study. First, we only measured the relationship between MPV and all-cause mortality and cardiovascular mortality, and didn’t evaluate relevant indicators such as mean platelet volume to platelet count ratio (MPV/PC), inflammatory cells and inflammatory factors, which to some extent didn’t accurately reflect the trend changes in blood parameters. Second, the lack of information on alcohol consumption, smoking, and other habits and living environments may also affect the level of lipids and ultimately the accuracy of the study results. At the same time, some unknown or uncollected factors may affect the accuracy of the study results. Finally, although we have controlled for many possible confounders to a certain extent, our results may still be biased by residual confounders and random errors. Therefore, our focus will be to monitor more relevant indicators, broaden the sample size and reduce confounding variables as much as possible in the future.

## Conclusion

High level MPV (MPV ≥ 10.2 fL) is independently related to all-cause mortality and cardiovascular mortality in patients treated with PD.

## Data Availability

The datasets used and/or analyzed during the current study available from the corresponding author (Email: zhibeng2501544@163.com) on reasonable request.

## References

[1] Akın F, Sert A, Arslan Ş. Mean platelet volume in children with hepatitis A. J Health Popul Nutr. 2016. 10.1186/s41043-016-0070-0 PMC5053354 27716426

[2] Gang L, Yanyan Z, Zhongwei Z, Juan D. Association between mean platelet volume and Hypertension incidence. Hypertens Res. 2017. 10.1038/hr.2017.30 28275234

[3] Vélez-Páez JL, Legua P, Vélez-Páez P, Irigoyen E, Andrade H, Jara A et al. Mean platelet volume and mean platelet volume to platelet count ratio as predictors of severity and mortality in sepsis. PLoS ONE. 2022. 10.1371/journal.pone.0262356 PMC8735631 34990467

[4] Omar M, Tanriverdi O, Cokmert S, Oktay E, Yersal O, Pilancı KN et al. Role of increased mean platelet volume (MPV) and decreased MPV/platelet count ratio as poor prognostic factors in Lung cancer. Clin Respiratory J. 2017. 10.1111/crj.12605 28026133

[5] Demir B, Abuzaid G. Association between Mean platelet volume, platelet Count, and distribution width with depth of Invasion in Oral Cancers. Ear, Nose & Throat Journal; 2022. 10.1177/01455613211032532 35411813

[6] Harrison P, Price J, Didembourg M, Johnson A, Baldwin S, Veronneau M et al. Feasibility of a mean platelet volume standard: an international council for standardization in hematology (ICSH) inter-laboratory study. Platelets. 2022. 10.1080/09537104.2022.2060956 35473564

[7] Ma S, Zhang J, Xu C, Da M, Xu Y, Chen Y, et al. Increased serum levels of cadmium are associated with an elevated risk of Cardiovascular Disease in adults. Environmental Science and Pollution Research; 2021. 10.1007/s11356-021-15732-2 34363163

[8] Korniluk A, Koper-Lenkiewicz OM, Kamińska J, Kemona H, Dymicka-Piekarska V. Mean platelet volume (MPV): New perspectives for an old marker in the course and prognosis of inflammatory conditions. Mediators of Inflammation; 2019. 10.1155/2019/9213074 PMC6501263 31148950

[9] Peng F, Li Z, Yi C, Guo Q, Yang R, Long H et al. Platelet index levels and cardiovascular mortality in incident peritoneal dialysis patients: a cohort study. Platelets. 2016. 10.1080/09537104.2016.1246716 27885913

[10] Ranjith MP, DivyaRaj R, Mathew D, George B, Krishnan MN. Mean platelet volume and cardiovascular outcomes in acute Myocardial Infarction. Heart Asia. 2016. 10.1136/heartasia-2015-010696 PMC4898625 27326224

[11] Lim WH, Chen JH, Minas K, Johnson DW, Ladhani M, Ooi E, et al. Sex disparity in cause-specific and all-cause mortality among Incident Dialysis patients. American Journal of Kidney Diseases; 2022. 10.1053/j.ajkd.2022.07.007 36029966

[12] Yardan T, Meric M, Kati C, Celenk Y, Atici AG. Mean platelet volume and mean platelet volume/platelet count ratio in risk stratification of Pulmonary Embolism. Medicina. 2016. 10.1016/j.medici.2016.03.001 27170484

[13] Faridi KF, Quispe R, Martin SS, Hendrani AD, Joshi PH, Brinton EA et al. Comparing different assessments of remnant lipoprotein cholesterol: the very large database of lipids. J Clin Lipidol. 2019. 10.1016/j.jacl.2019.06.001 31320236

[14] Camp RL, Dolled-Filhart M, Rimm DL. X-tile: a new bio-informatics tool for biomarker assessment and outcome-based cut-point optimization. Clin Cancer Res. 2004. 10.1158/1078-0432.CCR-04-0713 15534099

[15] Toida T, Sato Y, Ogata S, Wada A, Masakane I, Fujimoto S. Synergic impact of body Mass Index, Diabetes, and Age on Long-Term Mortality in Japanese Incident Hemodialysis patients: a Cohort Study on a large National Dialysis Registry. J Ren Nutr. 2019. 10.1053/j.jrn.2019.09.007 31812321

[16] Pusuroglu H, Cizgici AY, Demir AR, Uygur B, Ozal E (2021). Long-term Prognostic Value of Mean platelet volume in patients with Hypertension. Acta Cardiol Sin.

[17] Oh J-Y, Allison MA, Barrett-Connor E (2017). Different impacts of Hypertension and Diabetes Mellitus on all-cause and cardiovascular mortality in community-dwelling older adults: the Rancho Bernardo Study. J Hypertens.

[18] Han JS, Park KS, Lee MJ, Kim CH, Koo HM, Doh FM et al. Mean platelet volume is a prognostic factor in patients with acute kidney injury requiring continuous renal replacement therapy. J Crit Care. 2014. 10.1016/j.jcrc.2014.07.022 25138689

[19] Chu SG, Becker RC, Berger PB, Bhatt DL, Eikelboom JW, Konkle B et al. Mean platelet volume as a predictor of cardiovascular risk: a systematic review and meta-analysis. J Thromb Haemost. 2009. 10.1111/j.1538-7836.2009.03584.x PMC3755496 19691485

[20] Harutyunyan M, Torosoff M. Increased Mean platelet volume is Associated with decreased survival in cardiogenic shock patients receiving mechanical circulatory support. J Heart Lung Transplantation. 2021.

[21] Gao L, Shi Q, Li H, Guo Q, Yan J, Zhou L. Prognostic value of the combined variability of mean platelet volume and neutrophil percentage for short-term clinical outcomes of sepsis patients. Postgrad Med. 2020. 10.1080/00325481.2020.1823137 32912023

[22] Liu C, Zhou Y, He X, Ma J, Guo W, Dong B et al. Mean platelet volume/platelet count ratio predicts long-term mortality in patients with infective endocarditis. Biomark Med. 2020. 10.2217/bmm-2019-0258 32166976

[23] Guney Varal I, Dogan P, Acar Celik E, Güler Kazancı E. Mean platelet volume and Mean platelet Volume/Platelet count ratio are predictors of late-onset Sepsis in Preterm infants: a case-control study. Fetal and Pediatric Pathology; 2022. 10.1080/15513815.2022.2064574 35438038

[24] Martínez-Quintana E, Rodríguez-Hernández JL, Riaño-Ruiz M, Rodríguez-González F. Mean platelet volume and major adverse cardiovascular events in congenital Heart Disease patients. Clin Hemorheol Microcirc. 2019. 10.3233/CH-180471 31006669

[25] Wu F, Wang Q, Qiao Y, Yu Q, Wang F (2022). A new marker of short-term mortality and poor outcome in patients with acute ischemic Stroke: Mean platelet volume-to-lymphocyte ratio. Medicine.

[26] Kazdal H, Kanat A, Ozdemir B, Ozdemir V, Sulun Y, Guvercin AR et al. The relationship between mean platelet volume and the mortality of patient severe Head Trauma; first study. Int J Neurosci. 2022:1–8. 10.1080/00207454.2022.2130296 36172796

[27] Dratch A, Kleine C-E, Streja E, Soohoo M, Park C, Hsiung J-T et al. Mean Corpuscular Volume and Mortality in Incident Hemodialysis Patients. Nephron. 2019. 10.1159/000495726 PMC6465067 30625478

[28] Kim S, Molnar MZ, Fonarow GC, Streja E, Wang J, Gillen DL et al. Mean platelet volume and mortality risk in a national incident hemodialysis cohort. Int J Cardiol. 2016. 10.1016/j.ijcard.2016.06.074 PMC5929115 27400185

[29] Wu F, Wang Q, Qiao Y, Yu Q, Wang F (2022). A new marker of short-term mortality and poor outcome in patients with acute ischemic Stroke: Mean platelet volume-to-lymphocyte ratio. Med (Baltim).

[30] Choi A, Park I, Lee HS, Chung J, Kim MJ, Park YS (2022). Usefulness of complete blood count parameters to predict poor outcomes in cancer patients with febrile neutropenia presenting to the emergency department. Ann Med.

[31] Leonardi S, Gragnano F, Carrara G, Gargiulo G, Frigoli E, Vranckx P, et al. Prognostic implications of declining hemoglobin content in patients hospitalized with Acute Coronary syndromes. Journal of the American College of Cardiology; 2021. 10.1016/S0735-1097(21)01734-4 PMC8091415 33509394

[32] Guo E, Zhang C, Guo L, Song K, Wang G, Duan C (2021). Prognostic value of platelet distribution width and mean platelet volume in patients with laryngeal cancer. Future Oncol.

[33] Delago D, Knittelfelder O, Jakse G, Lukasiak K, Reinisch S, Renner W (2020). The decreased mean platelet volume is associated with poor prognosis in patients with oropharyngeal cancer treated with radiotherapy. Radiat Oncol.

[34] Detopoulou P, Panoutsopoulos GI, Mantoglou M, Michailidis P, Pantazi I, Papadopoulos S et al. Relation of Mean platelet volume (MPV) with Cancer: a systematic review with a focus on Disease Outcome on twelve types of Cancer. Curr Oncol. 2023. 10.3390/curroncol30030258 PMC10047416 36975471

[35] Marwa Abd ELRELA, El-Din Mohammed NA, Ghada Ahmed F, Yara Raafat S (2023). Mean platelet volume (MPV) and plasma lactate level in the diagnosis and prognosis of neonatal bacteremia.

[36] Kamath S, Blann AD, Lip GYH (2001). Platelet activation: assessment and quantification. Eur Heart J.

[37] GILES H, SMITH REA (1994). Platelet glycoprotein IIb-IIIa and size are increased in acute Myocardial Infarction. Eur J Clin Invest.

[38] Kim S, Molnar MZ, Fonarow GC, Streja E, Wang J, Gillen DL (2016). Mean platelet volume and mortality risk in a national incident hemodialysis cohort. Int J Cardiol.

[39] Sansanayudh N, Numthavaj P, Muntham D, Yamwong S, McEvoy M, Attia J (2015). Prognostic effect of mean platelet volume in patients with coronary artery Disease. Syst Rev meta-analysis.

[40] GROVE EL, HVAS AM, MORTENSEN SB, LARSEN SB (2011). Effect of platelet turnover on whole blood platelet aggregation in patients with coronary artery Disease. J Thromb Haemost.

